# The m^6^A reader MhYTP2 negatively modulates apple Glomerella leaf spot resistance by binding to and degrading *MdRGA2L* mRNA

**DOI:** 10.1111/mpp.13370

**Published:** 2023-06-27

**Authors:** Tianli Guo, Ru Bao, Zehua Yang, Xiaomin Fu, Liu Hu, Na Wang, Changhai Liu, Fengwang Ma

**Affiliations:** ^1^ State Key Laboratory of Crop Stress Biology for Arid Areas/Shaanxi Key Laboratory of Apple College of Horticulture, Northwest A&F University Yangling China

**Keywords:** Glomerella leaf spot, *Malus*, R genes, salicylic acid, YTH domain

## Abstract

Glomerella leaf spot (GLS), caused by the fungal pathogen *Colletotrichum fructicola*, significantly threatens apple production. Some resistances to plant disease are mediated by the accumulation of nucleotide‐binding site and leucine‐rich repeat (NBS‐LRR) proteins that are encoded by a major class of plant disease resistance genes (R genes). However, the R genes that confer resistance to GLS in apple remain largely unclear. *Malus hupehensis* YT521‐B homology domain‐containing protein 2 (MhYTP2) was identified as an *N* ^6^‐methyladenosine RNA methylation (m^6^A) modified RNA reader in our previous study. However, whether MhYTP2 binds to mRNAs without m^6^A RNA modifications remains unknown. In this study, we discovered that MhYTP2 exerts both m^6^A‐dependent and ‐independent functions by analysing previously obtained RNA immunoprecipitation sequencing results. The overexpression of *MhYTP2* significantly reduced the resistance of apple to GLS and down‐regulated the transcript levels of some R genes whose transcripts do not contain m^6^A modifications. Further analysis indicated that MhYTP2 binds to and reduces the stability of *MdRGA2L* mRNA. *MdRGA2L* positively regulates resistance to GLS by activating salicylic acid signalling. Our findings revealed that MhYTP2 plays an essential role in the regulation of resistance to GLS and identified a promising R gene, *MdRGA2L*, for use in developing apple cultivars with GLS resistance.

## Graphical abstract

The *Malus hupehensis* m^6^A reader MhYTP2 negatively modulates apple defence against Glomerella leaf spot by binding to and decreasing the stability of the mRNA of *MdRGA2L*, an R gene conferring resistance via salicylic acid signalling.

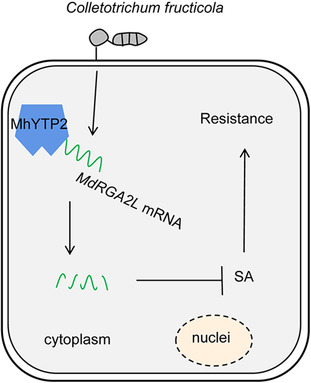

## 1 INTRODUCTION

Glomerella leaf spot (GLS), a fungal disease caused by *Colletotrichum fructicola*, causes fruit to have small light‐brown sunken lesions; these fruits do not increase in size over time and eventually drop, thereby reducing the yield and commodity value of crops (Velho et al., 2016; Wang et al., 2015). In addition, the leaves can be infected, which leads to early defoliation, weakening the tree and thus affecting fruit production the following year (Velho et al., 2015). The apple cultivars Golden Delicious, Gala, and Qinguan are highly susceptible to GLS (Liu et al., 2016).

So far, research on GLS in apple has focused on the isolation and identification of the pathogen (Moreira et al., 2019), identifying environmental conditions that affect its infection and spread (Wang et al., 2015), and determining control methods (Zhang et al., 2016). However, because there is no effective approach that is currently used to control GLS, our inability to protect susceptible apple varieties from *C. fructicola* infection results in significant loss of apple production (Wang et al., 2015). Therefore, clarifying the molecular mechanisms of disease resistance and breeding apple cultivars that are resistant to *C. fructicola* have become major objectives of China's apple breeding programme.

Plants have evolved complex mechanisms to induce defence reactions (Dangl & Jones, 2001; Ramirez‐Prado et al., 2018; Sharifi et al., 2018). Plant defence responses are mediated by pattern recognition receptors (PRRs) and cytoplasmic immune receptors (van Esse et al., 2020). The recognition of highly conserved pathogen‐associated molecular patterns (PAMP) by host cell surface PRRs is called pattern‐triggered immunity (PTI) (Parker et al., 2022). PTI triggers cytological and physiological responses that enable plants to resist pathogen infection, such as responses in nitric oxide, hormones, and expression of disease‐resistance genes (Xu et al., 2022; Yu et al., 2017). Use of the cytoplasmic immune receptors is a well‐known strategy that is characterized by specific interactions between a plant's resistance (R) gene and a pathogen's corresponding avirulence (Avr) gene leading to disease resistance (Ji et al., 2021). The largest group of R genes contain one or more nucleotide‐binding sites and leucine‐rich repeat (NBS‐LRR) domains that recognize virulence effectors, thus leading to effector‐triggered immunity (ETI) (Arya et al., 2014; McHale et al., 2006; Steinbrenner et al., 2015; Teixeira et al., 2014; Zhong et al., 2015). Plant disease resistance is characterized by the accumulation of NBS‐LRR proteins that are encoded by R genes. PTI and ETI, two major immune pathways in plants, amplify each other and act synergistically to ensure that plants can produce a lasting and strong immune response to pathogen invasion (Ngou et al., 2021; Yuan et al., 2021). In the ETI response, R genes can activate signalling pathways downstream of salicylic acid (SA) (Chen et al., 2021). SA plays an important role in defending plants against biotrophic pathogens (Samaradivakara et al., 2022). Additionally, SA levels in plants are mainly determined by the balance between SA biosynthesis and SA degradation. SA biosynthesis is mainly controlled by isochorismate synthase 1 (ICS1) (Seyfferth & Tsuda, 2014). SA hydroxylation is the main pathway mediating the degradation of SA. SA can be hydroxylated by the enzyme encoded by *Downy Mildew Resistant 6* (*DMR6*; Zhang et al., 2013, 2017). In apple (*Malus domestica*), *MdDMR6* is a homologue of *AtDMR6* and encodes SA hydroxylase, which converts SA into 2,5–dihydroxybenzoic acid, resulting in the inactivation of SA (Shan et al., 2021; Zhang et al., 2017).

The cellular response to environmental stress is usually mediated by RNA‐binding proteins (RBPs). Transgenic methods identified that MhYTP2, an RBP cloned from tea crab apple (*Malus hupehensis*), contains the YT521‐B homology (YTH) domain and plays important regulatory roles in drought stress, high salt stress, leaf senescence, and fruit development in apple (Liu et al., 2018; Wang, Guo, Sun, Wang, Shao, Liang, et al., 2017; Wang, Guo, Wang, Sun, Shao, Jia, et al., 2017; Wang, Guo, Wang, Sun, Shao, Liang, et al., 2017). Studies have also shown that MhYTP2 functions in basic RNA stability and powdery mildew (*Podosphaera leucotricha*) resistance (Guo et al., 2022). In the present study, we identified that MhYTP2 was also involved in apple resistance to GLS by directly binding to the mRNA of *MdRGA2L*, which encodes a coiled–coil (CC)‐NBS‐LRR protein, leading to the down‐regulation of *MdRGA2L* transcripts on *C. fructicola* infection. *MdRGA2L* enhanced resistance to GLS by activating SA signalling in apple. The identification of *MdRGA2L* provides a new in‐depth understanding of a molecular mechanism that underlies immunity to GLS in apple and could promote the molecular breeding of GLS‐resistant apple varieties.

## 2 RESULTS

### 2.1 *MhYTP2* reduces the resistance to GLS

**FIGURE 1:**
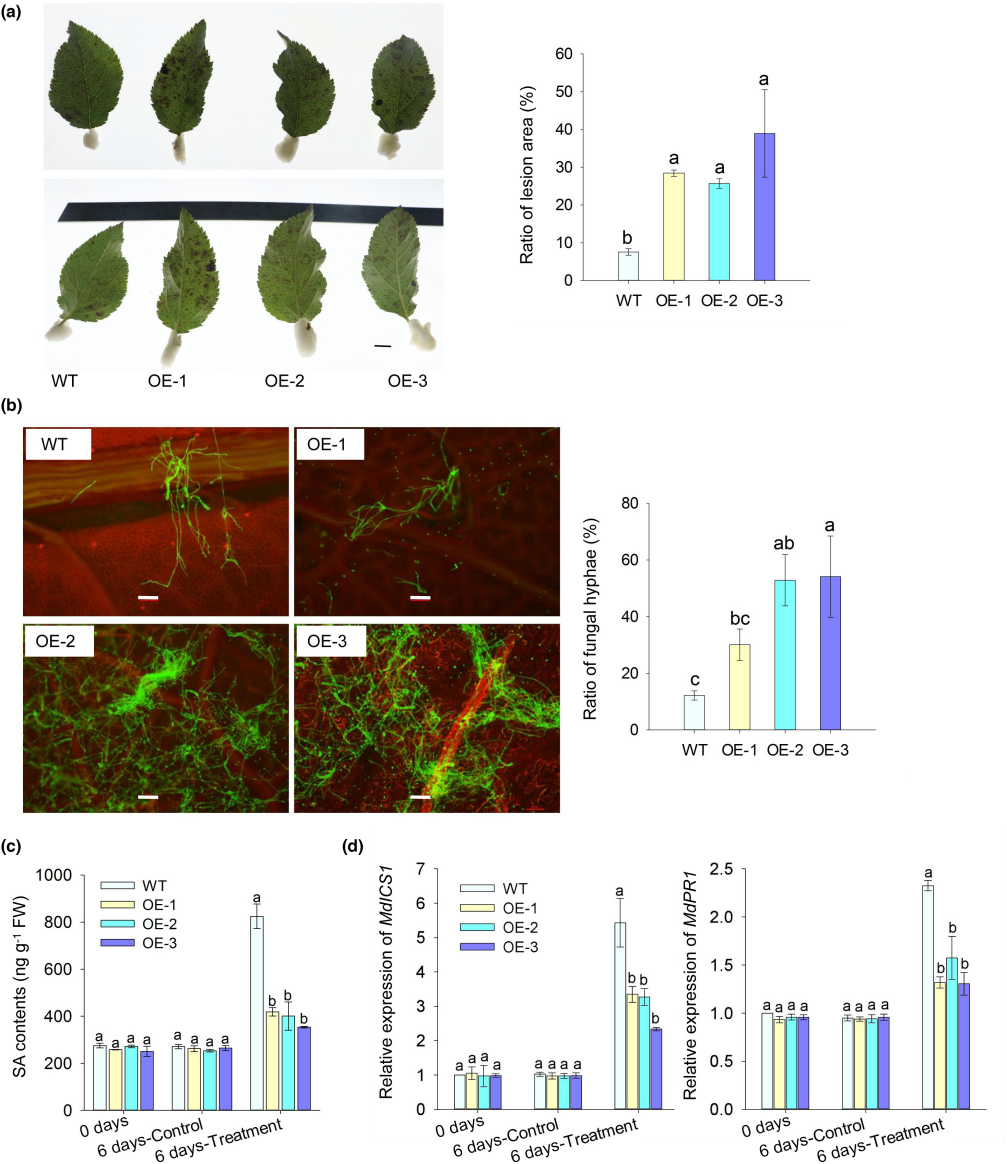
*MhYTP2* reduces resistance to Glomerella leaf spot (GLS). (a) Disease severity recorded and statistics of GLS susceptibility 6 days following inoculation with *Colletotrichum fructicola* of the overexpression transgenic lines (OE‐1, OE‐2, and OE‐3) and the wild‐type (WT) plants. Scale bars 1 cm. (b) The spread of the pathogen and its statistical summary in the inoculated apple leaves. Scale bars 1 mm. (c) The contents of salicylic acid (SA) in the transgenic lines and WT plants on the sixth day after inoculation with *C. fructicola* (6 days‐Treatment) or in the absence of *C. fructicola* (6 days‐Control). FW, fresh weight. (d) The transcript levels of *MdICS1* and *MdPR1* in the transgenic lines and the WT plants on the sixth day after inoculation with *C. fructicola* (6 days‐Treatment) or in the absence of *C. fructicola* (6 days‐Control). Data are presented as mean ± *SD*. Different letters indicate significant differences among the WT and transgenic plants on the same day of different treatments.

We occasionally found that three *35S::MhYTP2* overexpression lines (OE‐1, OE‐2, OE‐3) showed reduced resistance to GLS compared with the wild‐type (WT) plants under field conditions. To verify this discovery, mature leaves from the three *35S::MhYTP2* lines and the WT apple trees were tested for their resistance to GLS in the laboratory. At 6 days postinoculation (dpi), the *35S::MhYTP2* lines were more severely infected than the WT plants (Figure 1a,b). SA plays an important role in plant disease resistance. As shown in Figure 1c, inoculation with *C. fructicola* caused SA to accumulate in all the lines at 6 dpi compared with the group that was not inoculated. The SA content had no significant differences among the WT and *35S::MhYTP2* plants in the absence of *C. fructicola* inoculation. However, after 6 days of *C. fructicola* infection, the SA content was significantly reduced in the *35S::MhYTP2* lines compared with the WT plants (Figure 1c). The SA biosynthetic gene *ICS1* was consistently associated with SA content. The *pathogenesis‐related 1* (*PR1*) gene exhibited no significant differences between the WT and *35S::MhYTP2* plants in the absence of *C. fructicola* infection. At 6 dpi, the level of expression of *MdPR1* in the *35S::MhYTP2* lines was significantly lower than that in the WT plants (Figure 1d). These results suggest that the decreased resistance of 35S:*MhYTP2* apple lines to GLS could be related to the decrease in SA content.

### 2.2 MhYTP2 binds to a portion of the target mRNAs that is not m^6^A modified, including the mRNAs of R genes

**FIGURE 2:**
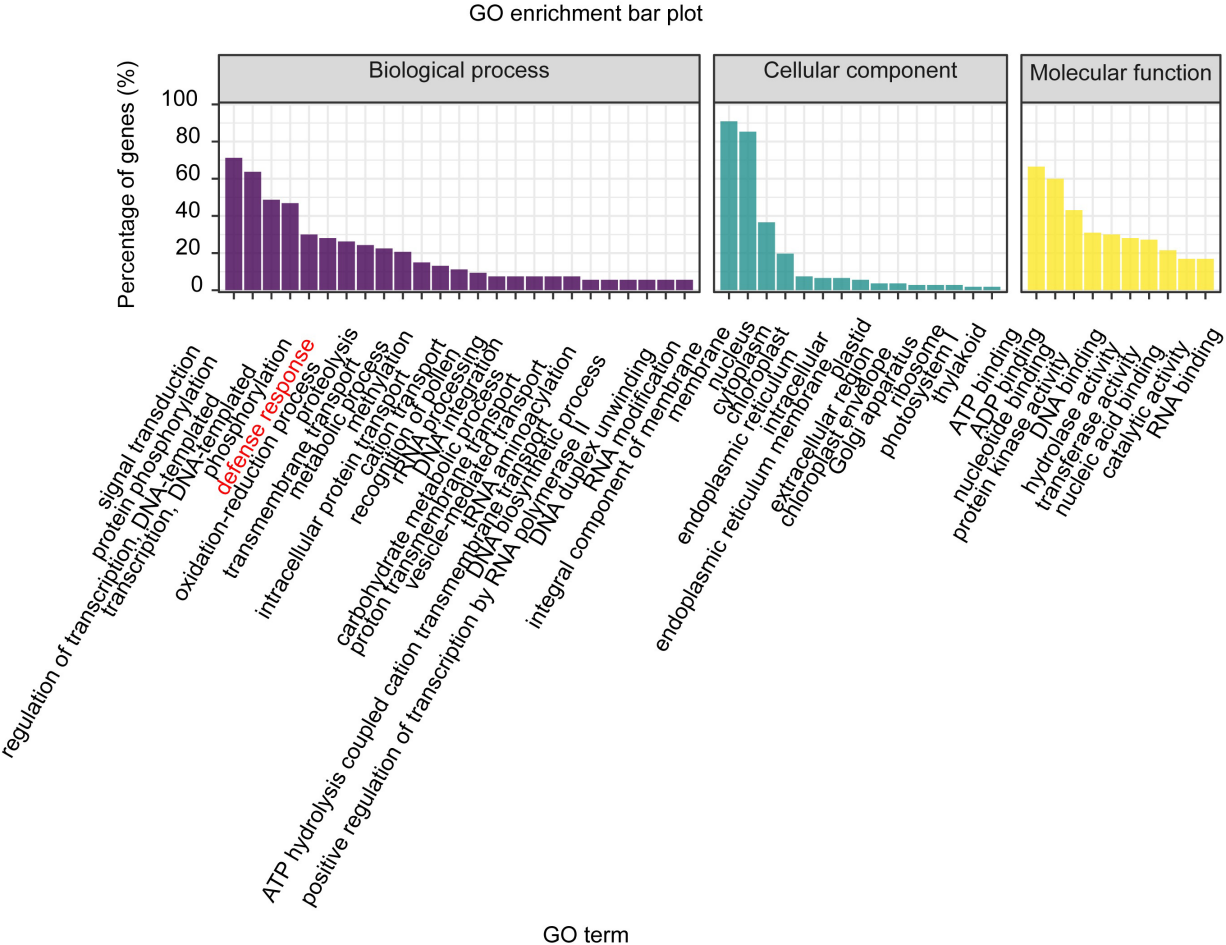
Gene Ontology (GO) enrichment analysis of the MhYTP2‐bound transcripts that were not modified by *N* ^6^‐methyladenosine RNA methylation.

We analysed the results of a previous RNA immunoprecipitation sequencing (RIP‐Seq) analysis (Guo et al., 2022). A Gene Ontology (GO) enrichment analysis of MhYTP2‐bound transcripts without *N* ^6^‐methyladenosine RNA methylation (m^6^A) modification was performed to gain functional insights into the role of MhYTP2 independent of m^6^A. The analysis showed that the MhYTP2‐bound transcripts without m^6^A modification were enriched in multiple signalling pathways and cellular processes (Figure 2). Because MhYTP2 negatively regulates GLS resistance, we focused on the genes that were enriched in the defence response pathways. Further analysis showed that MhYTP2 was bound to the mRNAs of 58 R genes that encode proteins with NBS‐LRR domains (Table S1).

### 2.3 MhYTP2 binds to *MdRGA2L* mRNA and reduces its stability

**FIGURE 3:**
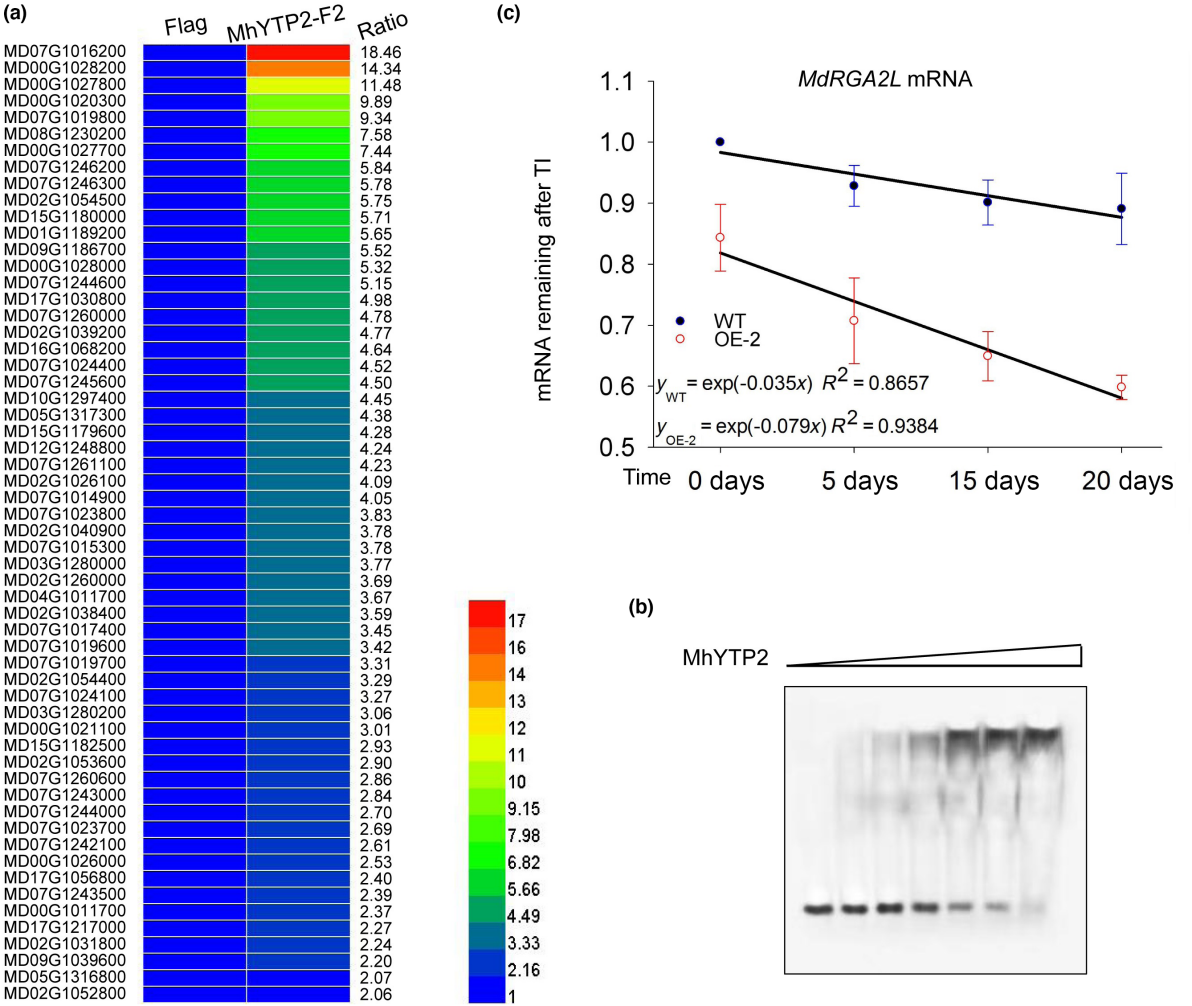
MhYTP2 binds to *MdRGA2L* (MD07G1016200) mRNA and reduces the stability of *MdRGA2L* mRNA. (a) Data from RNA immunoprecipitation sequencing shows that MhYTP2 binds to *MdRGA2L* mRNA. The ratio values on the right of the heatmap represent the ratio of the number of reads of MhYTP2‐F2 to that of FLAG. FLAG represents transgenic *35S::FLAG* apple callus and MhYTP2‐F2 represents transgenic *35S::MhYTP2‐FLAG* apple callus line 2. (b) Validation of MhYTP2 binding to *MdRGA2L* mRNA by electrophoretic mobility shift assay. (c) The mRNA lifetime of *MdRGA2L* in the transgenic line OE‐2 and the wild‐type (WT) plants. MhYTP2 nontarget *MdMDH* was used as the negative control. Data are represented as the mean ± *SD*.

*MdRGA2L* (MD07G1016200) is one of the 58 R genes bound by MhYTP2 that was enriched in defence response pathways, and it had the most numerous RIP‐Seq reads (Figure 3a). R genes play crucial roles in the regulation of plant resistance to biotic stress (Lee & Lee, 2005; Nandety et al., 2013). Thus, *MdRGA2L* was selected for further study. An electrophoretic mobility shift assay (EMSA) showed MhYTP2 binding to *MdRGA2L* mRNA (Figure 3b). Studies have demonstrated that MhYTP2 binds to and affects the stability of mRNA (Guo et al., 2022). We hypothesized that the role that MhYTP2 plays in the stability of *MdRGA2L* mRNA could affect the defence of apple against GLS, therefore the degradation rate of *MdRGA2L* was measured. The *MdRGA2L* transcript was rapidly degraded in the *35S::MhYTP2* line OE‐2 compared with the WT plants (Figure 3c), whereas the mRNA lifetimes of the negative control gene *MdMDH* in the *35S::MhYTP2* line OE‐2 and the WT plant were similar (Figure S1). This suggested that MhYTP2 promoted the degradation of *MdRGA2L* mRNA.

### 2.4 *MdRGA2L* is an R gene induced by *C. fructicola* and localized to the nucleus and cytosol

**FIGURE 4:**
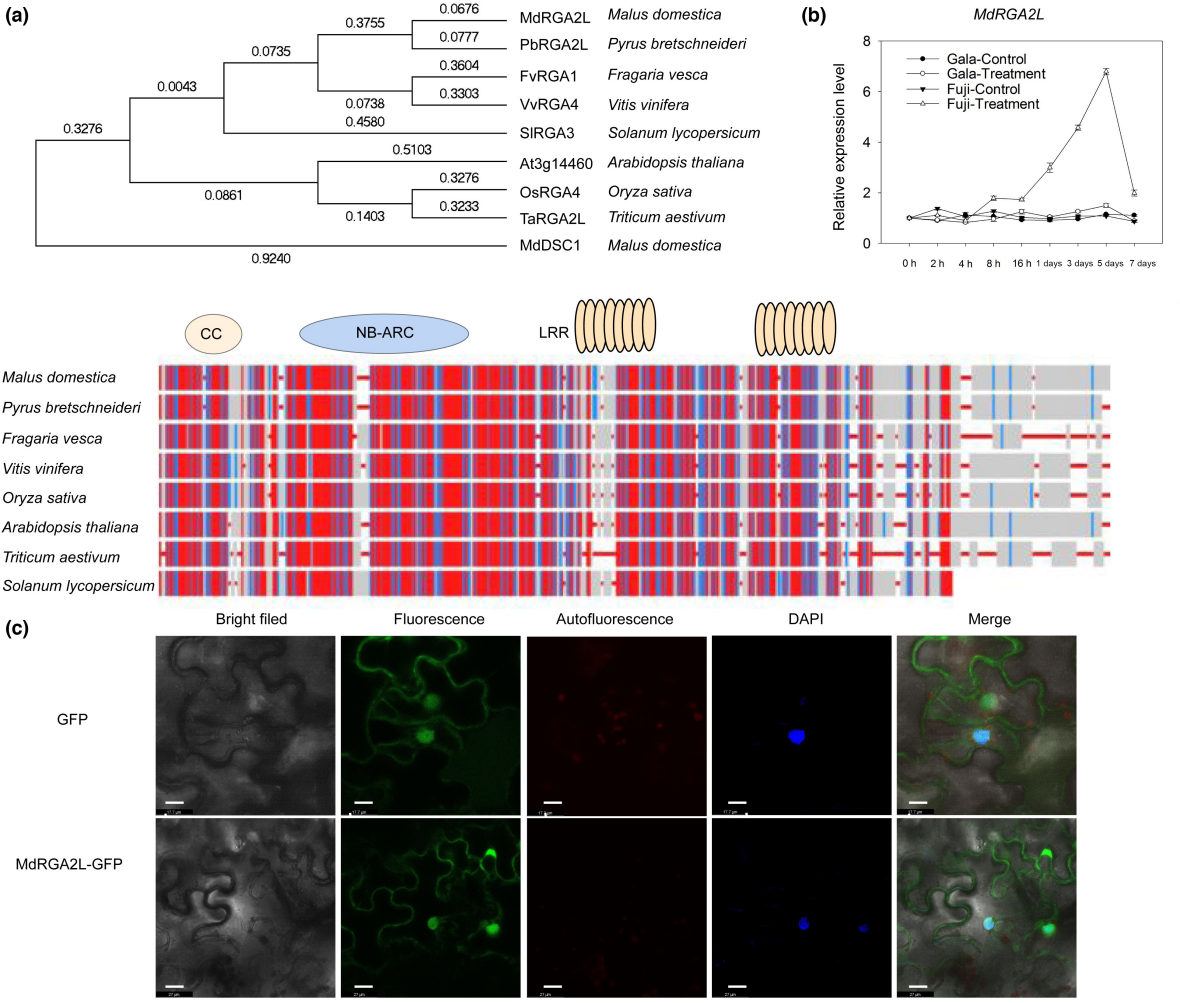
*MdRGA2L* is an R gene induced by *Colletotrichum fructicola* and localized to the nucleus and cytosol. (a) Phylogenetic and conserved structural domain analysis of MdRGA2L in plants. (b) The changes in transcript level of *MdRGA2L* during the progress of inoculation with *C*. *fructicola* of the Glomerella leaf spot (GLS)‐susceptible cultivar Gala and GLS‐resistant cultivar Fuji. (c) Subcellular localization of MdRGA2L. Upper panel, green fluorescent protein (GFP); lower panel, MdRGA2L‐GFP. Scale bars 16 μm. Data are represented as the mean ± *SD*. CC, coiled‐coil; DAPI, 4,6‐diamidino‐2‐phenylindole dihydrochloride; LRR, leucine‐rich repeat; NB‐ARC, nucleotide‐binding adaptor shared by Apaf1, certain R genes and CED4.

A phylogenetic analysis revealed that MdRGA2L shares high similarity with Chinese white pear (*Pyrus bretschneideri*) RGA2L (Figure 4a). We examined the changes in the transcript level of *MdRGA2L* between the GLS‐susceptible cultivar Gala and the GLS‐resistant cultivar Fuji under both control and *C. fructicola* inoculation conditions. As shown in Figure 4b, the transcript levels of *MdRGA2L* did not change significantly in the leaves of the uninoculated plants. On infection, the level of transcripts increased 6.3‐ and 1.3‐fold on the fifth day following inoculation with *C. fructicola* relative to that on the fifth day under the control conditions in Fuji and Gala cultivars, respectively (Table S2). This result suggests that *MdRGA2L* might be involved in apple resistance to *C. fructicola*. MdRGA2L was observed to be localized to the nucleus and cytosol (Figure 4c).

### 2.5 MhYTP2 decreased the transcript levels of *MdRGA2L* on *C. fructicola* infection

**FIGURE 5:**
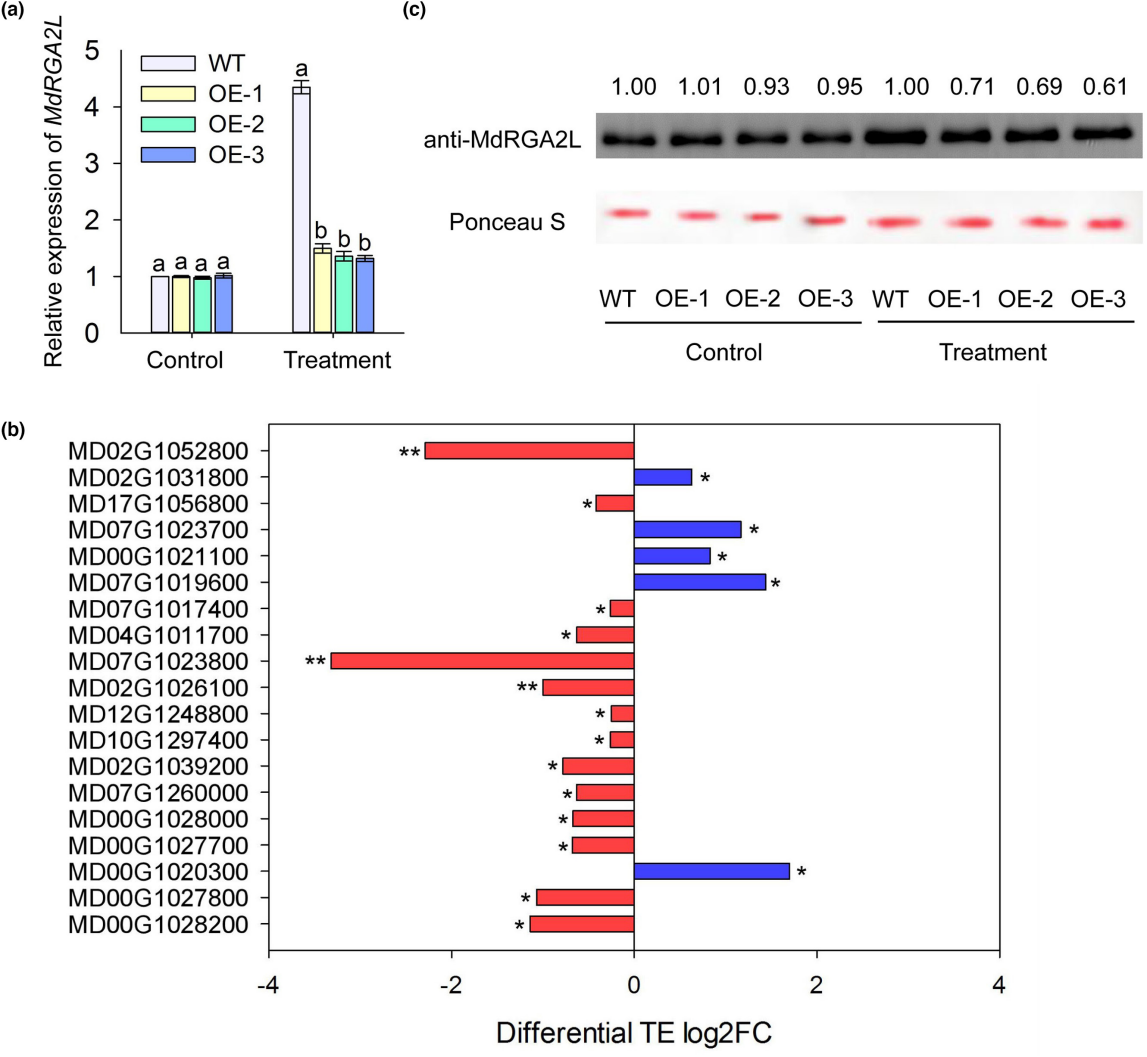
MhYTP2 decreased the transcript level of *MdRGA2L* after inoculation with *Colletotrichum fructicola*. (a) The transcript levels of *MdRGA2L* in the wild‐type (WT) and *35S::MhYTP2* overexpression lines (OE‐1, OE‐2, and OE‐3). Data are represented as the mean ± *SD*. Different letters indicate significant differences among the WT and transgenic plants on the same day of different treatments. (b) The R genes whose translation efficiency (TE) are affected by MhYTP2. (c) MdRGA2L protein expression in the *35S::MhYTP2* lines and the WT plants at the start and 6 days after inoculation with *C. fructicola*. Ponceau S staining shows the loading control. *p < 0.05, **p < 0.01.

We monitored the levels of expression of *MdRGA2L* in the WT and *35S::MhYTP2* plants. The transcript levels of *MdRGA2L* had no clear differences between the WT and *35S::MhYTP2* plants under control growth conditions. After inoculation with *C. fructicola* for 6 days, the transcript levels of *MdRGA2L* were up‐regulated in both the WT and *35S::MhYTP2* plants, but the *35S::MhYTP2* lines had an evident reduction compared with the WT plants (Figure 5a). MhYTP2 was previously shown to affect the translation efficiency of the bound mRNAs (Guo et al., 2022). To explore whether MhYTP2 affects the translation efficiency of the 58 R genes, we referred to the ribosome profiling (Ribo‐Seq) results of Guo et al. (2022), and 19 genes that had significant changes (fold change ≥2; *p* < 0.05) were found between the *35S::MhYTP2* lines and WT plants (Figure 5b). Among these 19 R genes, the translation efficiency of 14 significantly decreased and five increased. These data indicate that MhYTP2 is involved in the regulation of the translation efficiency of R genes.

We did not observe a significant change in the translation efficiency of *MdRGA2L* between the WT and *35S::MhYTP2* plants. We monitored the levels of MdRGA2L protein in comparison with the performance of WT plants. The MdRGA2L protein significantly decreased in *35S::MhYTP2* plants after inoculation with *C. fructicola*, but there was little difference among the WT and *35S::MhYTP2* plants under control growth conditions (Figure 5c).

These data indicate that MhYTP2 is involved in regulating the translation efficiency of R genes. MhYTP2 regulates the expression of *MdRGA2L* at the posttranscriptional level but not at the translational level.

### 2.6 *MdRGA2L* enhanced apple GLS resistance by activating SA signalling

**FIGURE 6:**
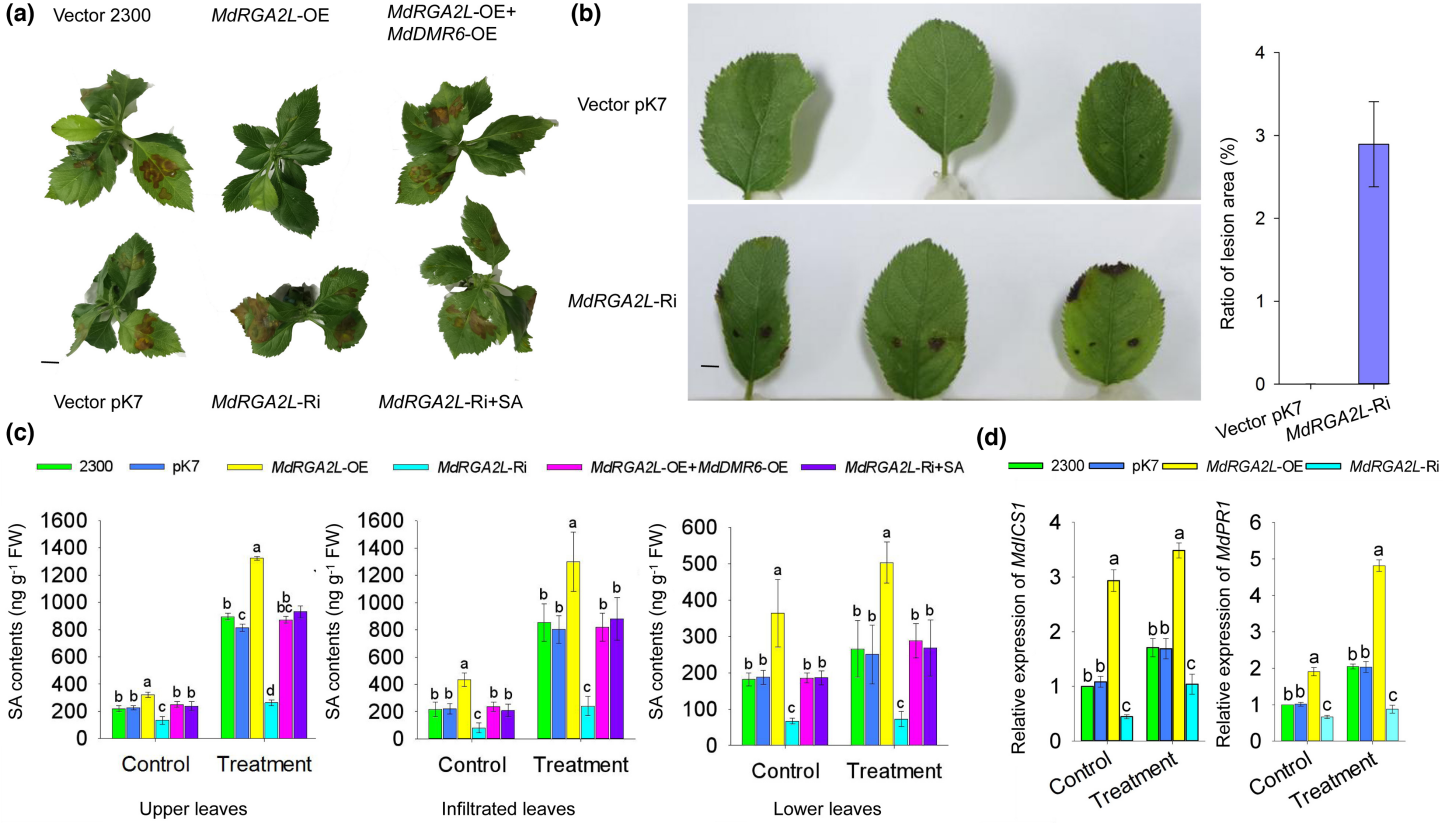
MdRGA2L enhanced the resistance of apple to Glomerella leaf spot (GLS) by activating the salicylic acid (SA) signalling pathway. (a) Disease severity was recorded 3 days after inoculation with *Colletotrichum fructicola* in the *MdRGA2L*‐OE, *MdRGA2L*‐Ri, empty vector (EV) control, *MdRGA2L*‐OE + *MdDMR6*‐OE, and *MdRGA2L*‐Ri + SA plants. Scale bars 1 cm. (b) Recorded disease severity and summary statistics of GLS susceptibility 3 days after inoculation with *C. fructicola* on Fuji *MdRGA2L‐*Ri and vector pK7‐expressing leaves. Scale bars 1 cm. (c) The contents of SA in the *MdRGA2L*‐OE, *MdRGA2L*‐Ri, EV control, *MdRGA2L*‐OE + *MdDMR6*‐OE, and *MdRGA2L*‐Ri + SA plants. FW, fresh weight. (d) The transcript levels of *MdICS1* and *MdPR1* in the *MdRGA2L*‐OE, *MdRGA2L*‐Ri, and EV control leaves. The EV controls are pCambia2300 (2300) and pK7WIWG2D (pK7). Data are represented as the mean ± *SD*. Different letters indicate significant differences among the wild‐type (WT) and transgenic plants on the same day of different treatments. OE, overexpression; Ri, RNA interference.

To explore the role of *MdRGA2L* in defending against GLS, its expression was modified by transforming an overexpression construct (*MdRGA2L*‐OE) or an antisense suppression construct (*MdRGA2L*‐Ri) into leaves of the *M. domestica* genotype GL‐3 (cv. Royal Gala) (Figure S2). When these 2‐day‐old empty vector (EV) and transgenic apple leaves were infected with *C. fructicola* for 3 days, the *MdRGA2L*‐OE apple leaves were strongly resistant to GLS compared with the EV control. In contrast, the *MdRGA2L*‐Ri apple leaves showed the opposite phenotype (Figure 6a). We also found that the *MdRGA2L*‐OE plants were more strongly resistant to GLS than the others when treated with *C. fructicola* for 6 days (Figure S3). To further confirm the role of *MdRGA2L* in the process of plant GLS resistance, we knocked down *MdRGA2L* in the young leaves of Fuji, a variety that is resistant to GLS (Figure S4). The leaves were inoculated with *C. fructicola* 2 days later, and we found that Fuji was no longer resistant to GLS (Figure 6b). These observations indicate that *MdRGA2L* has a substantial role in the defence of Fuji plants against GLS. In this experiment, we also observed that the contents of SA were modified by the alteration of *MdRGA2L* expression. The contents of SA and levels of *MdICS1* transcript showed a consistent trend with the level of *MdRGA2L* transcript. In addition, the levels of *MdPR1* transcript dramatically increased in the *MdRGA2L*‐OE plants and significantly decreased in the *MdRGA2L*‐Ri plants (Figure 6c,d). These results suggest that *MdRGA2L* may execute its role in GLS tolerance by regulating the contents of SA. To test this, *MdRGA2L* and the SA degradation gene *MdDMR6* were co‐expressed in apple leaves. When challenged with *C. fructicola* for 3 days, the apple leaves that overexpressed *MdRGA2L* and *MdDRM6* showed the same resistance to GLS as the EV control (Figure 6a). Moreover, to further determine whether *MdRGA2L* is involved in the SA signalling pathway, *MdRGA2L*‐Ri apple leaves (2 days after infiltration) were treated with 200 mg/L SA before they were inoculated with *C. fructicola*, *MdRGA2L*‐Ri apple leaves showed a similar phenotype as the EV control (Figure 6a). Collectively, these results indicate that *MdRGA2L* enhances the resistance of apple to GLS by activating SA signalling.

Surprisingly, we found that the whole plant exhibited resistance to GLS, including the leaves in which *MdRGA2L* was transiently overexpressed (infiltrated leaves) and the upper leaves and lower leaves that lacked transient *MdRGA2L* overexpression. Thus, we hypothesized that SA, which plays a critical role in systemic acquired resistance, was transported from the infiltrated leaves to the other leaves. To confirm this, we separately collected the infiltrated leaves in which *MdRGA2L* was transiently overexpressed and the upper and lower uninfiltrated leaves and measured their SA contents. The results showed that the levels of SA in all the samples of *MdRGA2L*‐OE plants tested were higher than those of the EV control, whereas the levels of SA in all the samples of *MdRGA2L*‐Ri plants tested were lower than those of the EV control (Figure 6c). These results suggest that *MdRGA2L* regulates the resistance of apple to GLS by promoting the biosynthesis of SA.

### 2.7 *MdRGA1* has no function in GLS resistance

**FIGURE 7:**
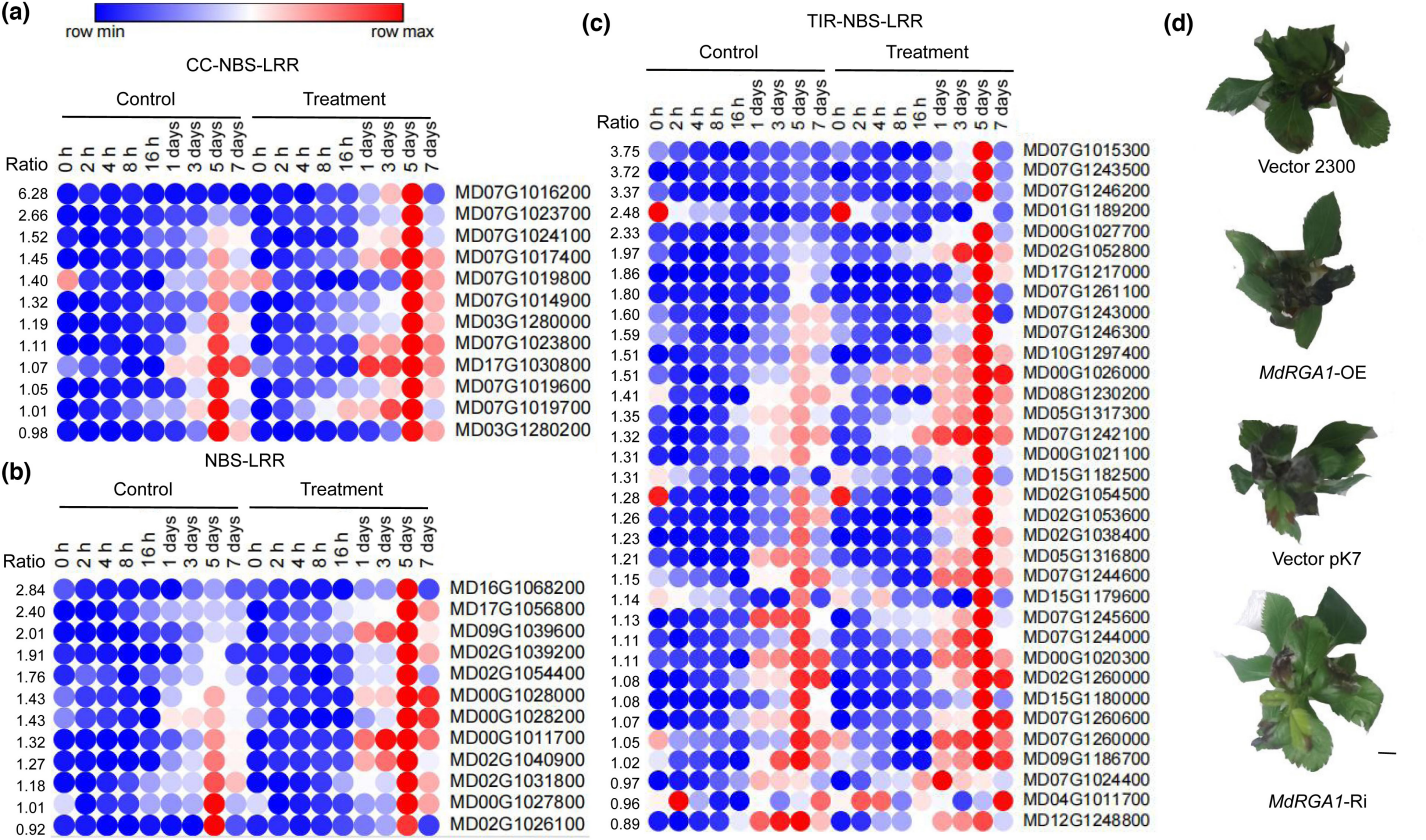
*MdRGA1* (MD07G1015300) has no function in Glomerella leaf spot (GLS) resistance. The changes in the transcript levels of genes encoding CC‐NBS‐LRR proteins (a), NBS‐LRR proteins (b), and TIR‐NBS‐LRR proteins (c) during the progression of inoculation with *Colletotrichum fructicola* in the GLS‐resistant cultivar Fuji. The ratio values on the left of the heatmap represent the number of transcripts of R genes in response to inoculation with *C. fructicola* in the GLS‐resistant cultivar Fuji at 5 days postinoculation compared with that of the control at day 5. (d) Disease severity recorded 3 days following the inoculation with *C. fructicola* of the *MdRGA1*‐OE, *MdRGA1*‐Ri, and empty vector (EV) plants. The EV controls are pCambia2300 (2300) and pK7WIWG2D (pK7). Scale bars 1 cm. CC‐NBS‐LRR, coiled‐coil nucleotide‐binding site leucine‐rich repeat; NBS‐LRR, nucleotide‐binding site leucine‐rich repeat; OE, overexpression; Ri, RNA interference; TIR‐NBS‐LRR, toll/interleukin‐1 receptor.

MhYTP2‐bound R genes can be primarily divided into three categories: CC‐NBS‐LRR proteins, NBS‐LRR proteins, and toll/interleukin‐1 receptor (TIR)‐NBS‐LRR proteins (Table S2). Among them, the CC‐NBS‐LRR proteins and TIR‐NBS‐LRR proteins were both more highly expressed when induced by *C. fructicola* than were the NBS‐LRR proteins (Figure 7a–c). We showed that MdRGA2L (a CC‐NBS‐LRR protein) plays a fundamental role in the resistance of apple to GLS. However, it is not known whether another MhYTP2 target, *MdRGA1* (MD07G1015300), the TIR‐NBS‐LRR gene that is the most significantly induced by GLS, plays a role in GLS resistance. To explore this, we overexpressed and knocked down *MdRGA1* in apple leaves and found that both types of leaves showed no difference in GLS resistance compared with the control plants (Figure 7d), implying that *MdRGA1* has no function in the resistance of apple plants to GLS.

## 3 DISCUSSION

GLS induces yield losses that have ranged from 30% to 70% over the past decade in China (Zhang et al., 2008). GLS weakens the fruit tree vitality, causes fruit necrosis, and reduces fruit quality owing to the defoliation of leaves (Shan et al., 2021; Velho et al., 2016; Wang et al., 2015). Additionally, GLS develops rapidly and is difficult to prevent (Lv et al., 2022; Shang, Wang, et al., 2020; Zhang et al., 2016). At present, there is no effective way to control GLS outbreaks. The extensive application of a variety of fungicides has not effectively controlled the disease (Wang et al., 2015). Previous studies explored the mechanisms controlling apple defence against GLS. However, the molecular mechanisms that underlie the resistance of apple to GLS remain poorly understood.

Most of the studies on the regulation of disease resistance using m^6^A readers were performed in mammals. The RNA m^6^A reader YTHDF2 targets AXIN1 and subsequently affects its stability to promote proliferation and metastasis of lung adenocarcinoma cells (Li et al., 2021). YTHDF2 promotes the degradation of mRNA and cancer progression by increasing its binding affinity to m^6^A‐modified mRNAs (Hou et al., 2021). The nuclear m^6^A reader YTHDC1 plays a critical role in leukemogenesis by regulating MCM complex‐mediated DNA replication (Sheng et al., 2021). The nuclear m^6^A reader IGF2BP enhances mRNA stability and translation, which enables the development of cancer (Huang et al., 2018). However, the available functional data are scarce in plants. MhYTP2 positively regulates resistance against powdery mildew by binding to and affecting the stability of *MdMLO19* and *MdPAL1* mRNAs in apple as an m^6^A reader (Guo et al., 2022). To our knowledge, this study is the first to report that MhYTP2, a YTH‐domain‐containing RNA‐binding protein, negatively modulates the defence of apple against GLS by regulating the stability of *MdRGA2L* mRNA in an m^6^A‐independent manner.

NBS‐LRR proteins can be divided into two classes according to their N‐terminal domains, including the toll/interleukin‐1 receptor (TIR) domain for NBS‐LRR proteins (TNL) and the coiled‐coil (CC) domain for NBS‐LRR proteins (CNL) (Funk et al., 2018). In the present study, we discovered that *MdRGA2L*, which encodes a CNL protein, is a functional R gene in apple. Its overexpression substantially reduced the susceptibility to GLS, whereas its inactivation substantially increased the susceptibility to GLS. The overexpression of *MhYTP2* accelerated the degradation of *MdRGA2L* mRNA, leading to a reduction in resistance against GLS. We previously demonstrated that MhYTP2 positively regulates apple resistance against drought, salt, and powdery mildew when overexpressed (Guo et al., 2022; Liu et al., 2018; Wang, Guo, Sun, Wang, Shao, Liang, et al., 2017; Wang, Guo, Wang, Sun, Shao, Liang, et al., 2017). How can this GLS susceptibility weakness of MhYTP2 be overcome? Considering that MhYTP2 directly binds to *MdRGA2L* mRNA and accelerates its degradation, the mutation of *MdRGA2L* at suitable sites with genome editing technology may help its mRNA avoid recognition by MhYTP2, which could be used to maintain GLS resistance.

TNLs have been reported as resistance genes in *Arabidopsis thaliana* (Gassmann et al., 1999), grape (*Vitis vinifera*) (Li et al., 2017), and rice (*Oryza sativa*) (Zhao et al., 2016). In apple plants, the apple scab (*Venturia inaequalis*) resistance locus *Rvi15* (*Vr2*) has been found to contain three TNL genes (Galli et al., 2010). The TNL gene *MdTNL1* is the key factor that regulates the resistance of apple to GLS (Lv et al., 2022). In the present study, we determined that the TNL gene *MdRGA1* has no function in the resistance of apple to GLS.

Some studies showed that deletion of the N‐terminal domain of NBS‐LRRs could not induce plant immunity. However, the expression of some N‐terminal domains of NBS‐LRRs alone is sufficient to elicit a hypersensitive response (Collier et al., 2011). In the present study, our findings did not reveal whether the CC domain of MdRGA2L induces plant immunity or not. In addition, whether or not the CC domain of MdRGA2L is involved in GLS resistance needs to be determined in the future.

SA plays an important role in defending plants against biotrophic pathogens (Samaradivakara et al., 2022). Exogenous application of SA can result in strong resistance to GLS (Zhang et al., 2016). Plants immediately stimulate their own PTI and ETI responses to respond when they perceive pathogens, and both of these immune responses are related to SA. PTI can promote the expression of *ICS1*, which leads to the gradual accumulation of SA (Wildermuth et al., 2001). In the ETI reaction, the recognition of R genes in host cells with effector proteins secreted by pathogens can activate signalling pathways downstream of SA (Chen et al., 2021).

**FIGURE 8:**
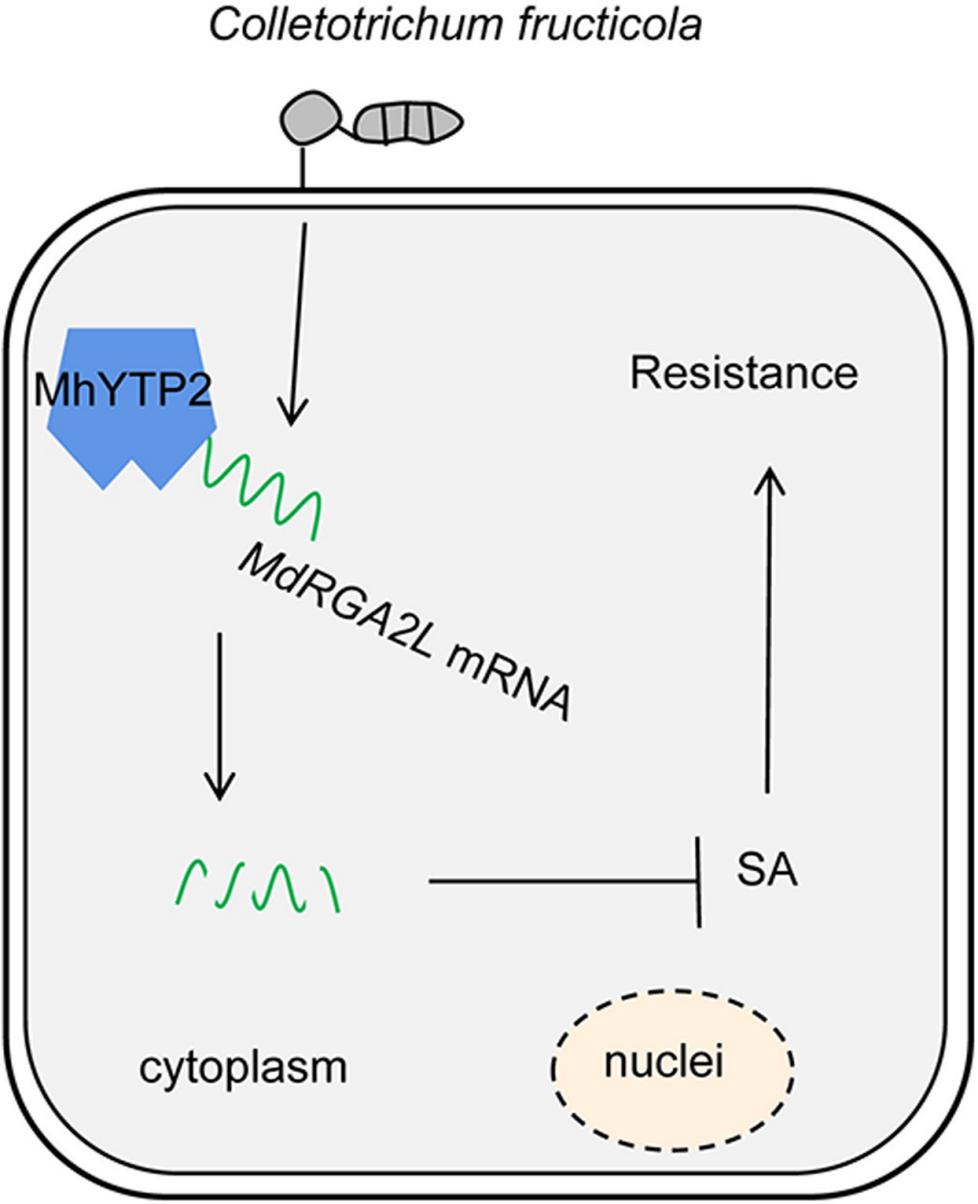
The proposed model describes how the m^6^A reader protein MhYTP2 regulates apple resistance to Glomerella leaf spot. SA, salicylic acid.

Based on these experimental results, we summarized the model of MhYTP2 regulating plant resistance to GLS (Figure 8). In this model, *C. fructicola* infection up‐regulates the expression of *MdRGA2L*. MhYTP2 directly binds to the mRNA of *MdRGA2L* and decreases its stability, which reduces the expression level of *MdRGA2L* after inoculation with *C. fructicola*. *MdRGA2L* directly increases the SA content by promoting the expression of the SA synthesis‐related gene *ICS1*. Subsequently, the increase in SA induces systemic acquired resistance. Thus, *MdRGA2L* has been identified to have substantial potential in breeding excellent apple varieties that are resistant to GLS.

## 4 EXPERIMENTAL PROCEDURES

### 4.1 Plant materials

The WT and *MhYTP2* transgenic *M. domestica* ‘Royal Gala’ plants were the same as those previously used in drought experiments (Liu et al., 2018).

For *MdRGA2L* and *MdRGA1* GLS resistance assays, tissue‐cultured WT plants and transgenic culture conditions are as previously described (Guo et al., 2019). The tobacco (*Nicotiana tabacum*) plants were grown under a 16 h:8 h light:dark photoperiod at 21°C in a growth chamber.

### 4.2 Fungal culture, leaf infection, and disease incidence statistics

The cultures of the pathogenic fungus *C. fructicola* were obtained and maintained as previously described (Li et al., 2023; Lv et al., 2022; Shang, Liang, et al., 2020), and 10^6^ cfu/mL inoculum was used to inoculate the plants (Figure S5). Each inoculum was quantified by microscopy (CX31RTSF; Olympus) and used to inoculate detached apple leaves by spraying them with a spore suspension.

Mature leaves were collected from trees growing in the field and disinfected with 75% (vol/vol) ethanol, then the bottoms of their petioles were wrapped with wet cotton balls prior to culture at 75% humidity and 25°C. Six days after inoculation, the necrotic lesions of each leaf were visually observed and recorded. Three biological replicates were performed on about 30 leaves from different plants.

The expression vectors were constructed by inserting the open reading frames of *MdRGA2L, MdRGA1*, or *MdDRM6* into the pCambia2300 vector. The vectors pHellsgate2 and pK7WIWG2D were used as RNAi‐mediated vectors to silence *MdRGA2L* or *MdRGA1* as described by Zhou et al. (2017). These vectors were then transferred to *Agrobacterium tumefaci* *ens* EHA105 to mediate transient expression in apple leaves. The functions of MdRGA2L and MdRGA1 in apple leaves were analysed using the *Agrobacterium*‐mediated transient expression method (Zhang et al., 2018). Three days after agro‐infiltration, six apple leaves were collected from different plants to detect the relative transcription levels of target genes and those of the genes related to the defence response in apple. The experiments were conducted in triplicate.

The areas of leaves with disease symptoms and the areas covered by GLS fungus were analysed using ImageJ software (NIH).

### 4.3 Microscopy

Fungal hyphae were stained with WGA‐AF 488 (Molecular Probes). The plant cells were visualized using propidium iodide (Sigma‐Aldrich). The sample processing and imaging observations were conducted as previously described (Guo et al., 2022). To obtain fluorescence of the fungal hyphae and plant cells, excitation wavelengths of 488 nm and 561 nm were used, respectively. Fluorescence was observed using a confocal laser scanning microscope (TCS‐SP8 SR; Leica) at 800 V.

### 4.4 Phylogenetic tree construction and subcellular localization analysis

We used BLASTP to identify protein sequences in other species that were homologous to MdRGA2L. A phylogenetic tree was constructed using MEGA 5 software (http://www.megasoftware.net/) (Jiang et al., 2019).

Subcellular localization was performed as previously described (Guo et al., 2020).

### 4.5 Western blot analysis

A western blot analysis was performed as previously described (Guo et al., 2022).

### 4.6 mRNA stability assay, RIP‐Seq, Ribo‐Seq, and EMSA

The mRNA stability was assayed as previously described (Guo et al., 2022). The data analysed in RIP‐Seq and Ribo‐Seq were derived from previous research results (Guo et al., 2022).

The EMSA was performed as previously described (Guo et al., 2022).

### 4.7 RNA extraction and gene expression analysis

The total RNA of the collected samples was isolated using a spin column plant total RNA purification kit (Shanghai Sangon Biotech). The isolated RNA (5 μg) was used for synthesizing first‐strand cDNA with MightyScript first‐strand cDNA synthesis master mix (Yeasen). Reverse transcription‐quantitative real‐time PCR (RT‐qPCR) was conducted as previously described (Guo et al., 2022) using the ChamQ SYBR qPCR Master Mixture (Vazyme Biotech). *Actin* (XM_008344381) was used as the reference gene and fold changes in gene expression were calculated using the 2^−∆∆Ct^ method (Kim et al., 2009; Livak & Schmittgen, 2001). All the experiments comprised three biological replicates. The primers are listed in Text S1.

### 4.8 SA quantification

The SA content was extracted and detected by liquid chromatography‐tandem mass spectrometry (LC–MS/MS; AB Sciex) in the Horticulture Science Research Center at the College of Horticulture at Northwest A&F University, Xianyang, China. The treatment and assay procedures for the samples followed previously reported methods for the determination of plant hormones (Guo et al., 2019). The content was calculated based on a standard curve. Each measurement was repeated three times.

### 4.9 Gene ontology analyses

The clean reads obtained were aligned to the apple reference genome GDDH13 (https://iris.angers.inra.fr/gddh13/the‐apple‐genome‐downloads.html) by HISAT2 (Kim et al., 2015). The reads mapped to each gene were used by FeatureCounts (Liao et al., 2014) for calculation. The differentially expressed genes identified by RIP‐Seq were further subjected to GO analysis using the agriGO database v. 2.0 (http://systemsbiology.cau.edu.cn/agriGOv2/).

### 4.10 Statistical analysis

The data were subjected to a one‐way analysis of variance (ANOVA), and the differences among treatment means were assessed by Tukey's test (*p* < 0.05). SPSS statistical software was used to analyse the data (IBM Inc.).

## CONFLICT OF INTEREST STATEMENT

The authors declare no conflicts of interest.

## Supporting information


**FIGURE S1.** The mRNA lifetime of *MdMDH* in the transgenic line OE‐2 and the wild‐type (WT) plants. Data are represented as the mean ± *SD*

**FIGURE S2.** Confirmation of transgenic apple *MdRGA2L*‐OE, *MdRGA2L*‐Ri, and EV control. The expression levels of *MdRGA2L* in the *MdRGA2L*‐OE, *MdRGA2L*‐Ri, and EV apple leaves. The EV control included vector 2300 and vector pK7. Vector 2300 is short for pCambia2300 and vector pK7 is short for pK7WIWG2D. Data are represented as the mean ± *SD*. Different letters indicate significant differences among different types of plants of different treatments. EV, empty vector; OE, overexpression; Ri, RNA interference

**FIGURE S3.** Disease severity recorded at 6 days postinoculation with *Colletotrichum fructicola* of the *MdRGA2L*‐OE, *MdRGA2L*‐Ri, EV control, *MdRGA2L*‐OE + *MdDMR6*‐OE, and *MdRGA2L*‐Ri + SA plants. The EV control included vector 2300 and vector pK7. Vector 2300 is short for pCambia2300 and vector pK7 is short for pK7WIWG2D. Scale bar 1 cm. EV, empty vector; OE, overexpression; Ri, RNA interference

**FIGURE S4.** Confirmation of transgenic Fuji apple leaves transformed with *MdRGA2L*‐Ri and vector pK7. The expression levels of *MdRGA2L* in the Fuji *MdRGA2L‐*Ri and vector pK7 control leaves. Data are represented as the mean ± *SD*. The empty vector pK7 is short for pK7WIWG2D. Ri, RNA interference

**FIGURE S5.** Images of *Colletotrichum fructicola* and its spores

**TABLE S1.** The 58 R genes identified by RIP‐Seq. RIP‐Seq, RNA immunoprecipitation sequencing

**TABLE S2.** Changes in the levels of expression of the R genes during the progression of inoculation with *Colletotrichum fructicola* of the GLS‐resistant cultivar Fuji. GLS, glomerella leaf spot

**TEXT S1.** RNA probe and primers used

## Data Availability

The data that support the findings of this study are available from the corresponding author upon reasonable request.
