# Supplementary material for: The m6A reader MhYTP2 negatively modulates apple Glomerella leaf spot resistance by binding to and degrading MdRGA2L mRNA
Source: Mol Plant Pathol. 2023 Jun 27;24(10):1287–99. doi: 10.1111/mpp.13370 (PMC10502827; doi:10.1111/mpp.13370)
Supplement: Supplementary file 1 — FIGURE S1. The mRNA lifetime of MdMDH in the transgenic line OE‐2 and the wild‐type (WT) plants. Data are represented as the mean ± SD [file MPP-24-1287-s007.docx]

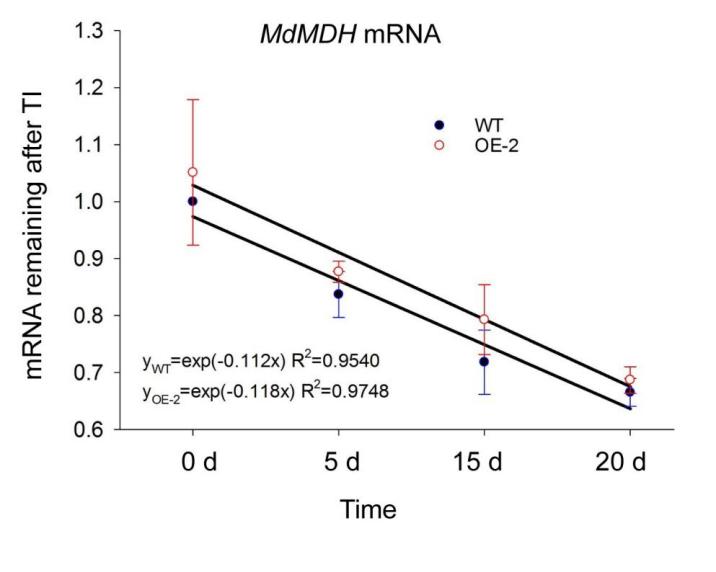


**FIGURE S1** The mRNA lifetime of *MdMDH* in the transgenic line OE-2 and the wild type (WT) plants. Data are represented as the means ± SD.
