# Supplementary material for: The m6A reader MhYTP2 negatively modulates apple Glomerella leaf spot resistance by binding to and degrading MdRGA2L mRNA
Source: Mol Plant Pathol. 2023 Jun 27;24(10):1287–99. doi: 10.1111/mpp.13370 (PMC10502827; doi:10.1111/mpp.13370)
Supplement: Supplementary file 2 — FIGURE S2. Confirmation of transgenic apple MdRGA2L‐OE, MdRGA2L‐Ri, and EV control. The expression levels of MdRGA2L in the MdRGA2L‐OE, MdRGA2L‐Ri, and EV apple leaves. The EV control included vector 2300 and vector pK7. Vector 2300 is short for pCambia2300 and vector pK7 is short for pK7WIWG2D. Data are represented as the mean ± SD. Different letters indicate significant differences among different types of plants of different treatments. EV, empty vector; OE, overexpression; Ri, RNA interference [file MPP-24-1287-s008.docx]

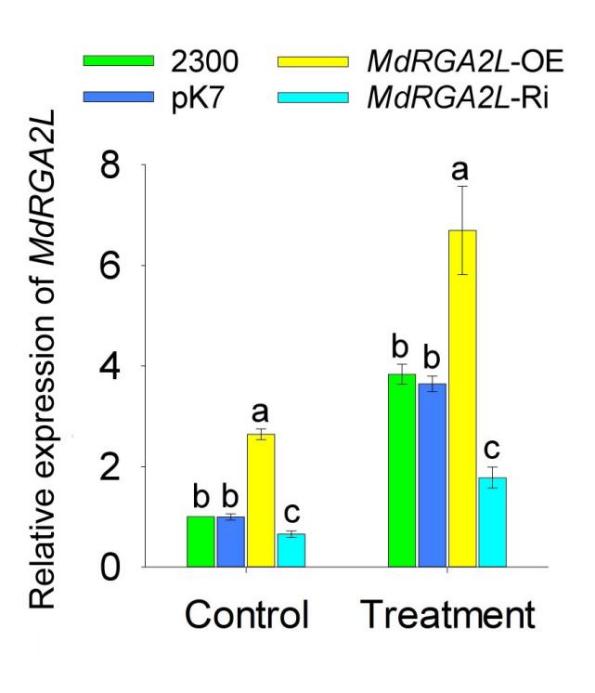


**FIGURE S2** Confirmation of transgenic apple *MdRGA2L*-OE, *MdRGA2L*-Ri, and EV control. The expression levels of *MdRGA2L* in the *MdRGA2L*-OE, *MdRGA2L*-Ri, and EV-expressed apple leaves. The EV control includes vector 2300 and vector pK7. Vector 2300 is short for pCambia2300, and vector pK7 is short for pK7WIWG2D. Data are represented as the means ± SD. Different letters indicate significant differences among different types of plants of different treatments. EV, empty vector; OE, overexpression; Ri, RNA interference.
