# Supplementary material for: The m6A reader MhYTP2 negatively modulates apple Glomerella leaf spot resistance by binding to and degrading MdRGA2L mRNA
Source: Mol Plant Pathol. 2023 Jun 27;24(10):1287–99. doi: 10.1111/mpp.13370 (PMC10502827; doi:10.1111/mpp.13370)
Supplement: Supplementary file 3 — FIGURE S3. Disease severity recorded at 6 days postinoculation with Colletotrichum fructicola of the MdRGA2L‐OE, MdRGA2L‐Ri, EV control, MdRGA2L‐OE + MdDMR6‐OE, and MdRGA2L‐Ri + SA plants. The EV control included vector 2300 and vector pK7. Vector 2300 is short for pCambia2300 and vector pK7 is short for pK7WIWG2D. Scale bar 1 cm. EV, empty vector; OE, overexpression; Ri, RNA interference [file MPP-24-1287-s003.docx]

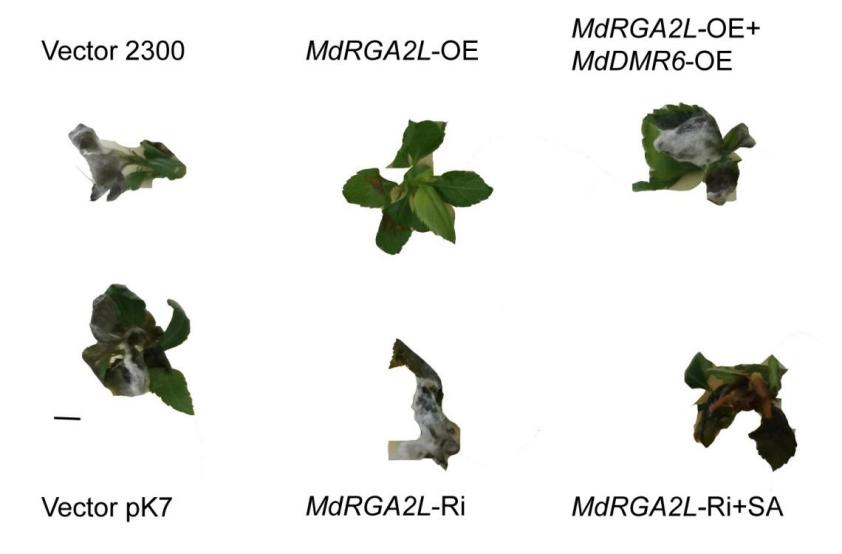


**FIGURE S3** Disease severity recorded at 6 dpi with *Colletotrichum fructicola* of the *MdRGA2L*-OE, *MdRGA2L*-Ri, EV control, *MdRGA2L*-OE+*MdDMR6*-OE, and *MdRGA2L*-Ri +SA –treated plants. The EV control includes vector 2300 and vector pK7. Vector 2300 is short for pCambia2300, and vector pK7 is short for pK7WIWG2D. Bar = 1 cm. EV, empty vector; OE, overexpression; Ri, RNA interference.
