# Supplementary material for: The m6A reader MhYTP2 negatively modulates apple Glomerella leaf spot resistance by binding to and degrading MdRGA2L mRNA
Source: Mol Plant Pathol. 2023 Jun 27;24(10):1287–99. doi: 10.1111/mpp.13370 (PMC10502827; doi:10.1111/mpp.13370)
Supplement: Supplementary file 4 — FIGURE S4. Confirmation of transgenic Fuji apple leaves transformed with MdRGA2L‐Ri and vector pK7. The expression levels of MdRGA2L in the Fuji MdRGA2L‐Ri and vector pK7 control leaves. Data are represented as the mean ± SD. The empty vector pK7 is short for pK7WIWG2D. Ri, RNA interference [file MPP-24-1287-s004.docx]

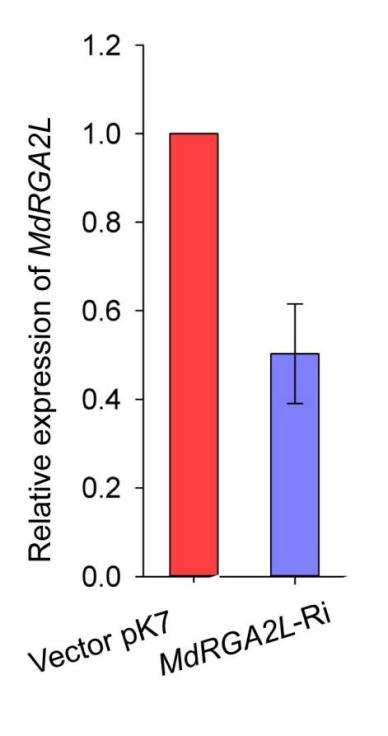


**FIGURE S4** Confirmation of transgenic ‘Fuji’ apple leaves *MdRGA2L*-Ri and vector pK7. The expression levels of *MdRGA2L* in the ‘Fuji’ *MdRGA2L-*Ri and vector pK7 control expressed leaves. Data are represented as the means ± SD. The empty vector pK7 is short for pK7WIWG2D. Ri, RNA interference.
