# Supplementary material for: The m6A reader MhYTP2 negatively modulates apple Glomerella leaf spot resistance by binding to and degrading MdRGA2L mRNA
Source: Mol Plant Pathol. 2023 Jun 27;24(10):1287–99. doi: 10.1111/mpp.13370 (PMC10502827; doi:10.1111/mpp.13370)
Supplement: Supplementary file 5 — FIGURE S5. Images of Colletotrichum fructicola and its spores [file MPP-24-1287-s001.docx]

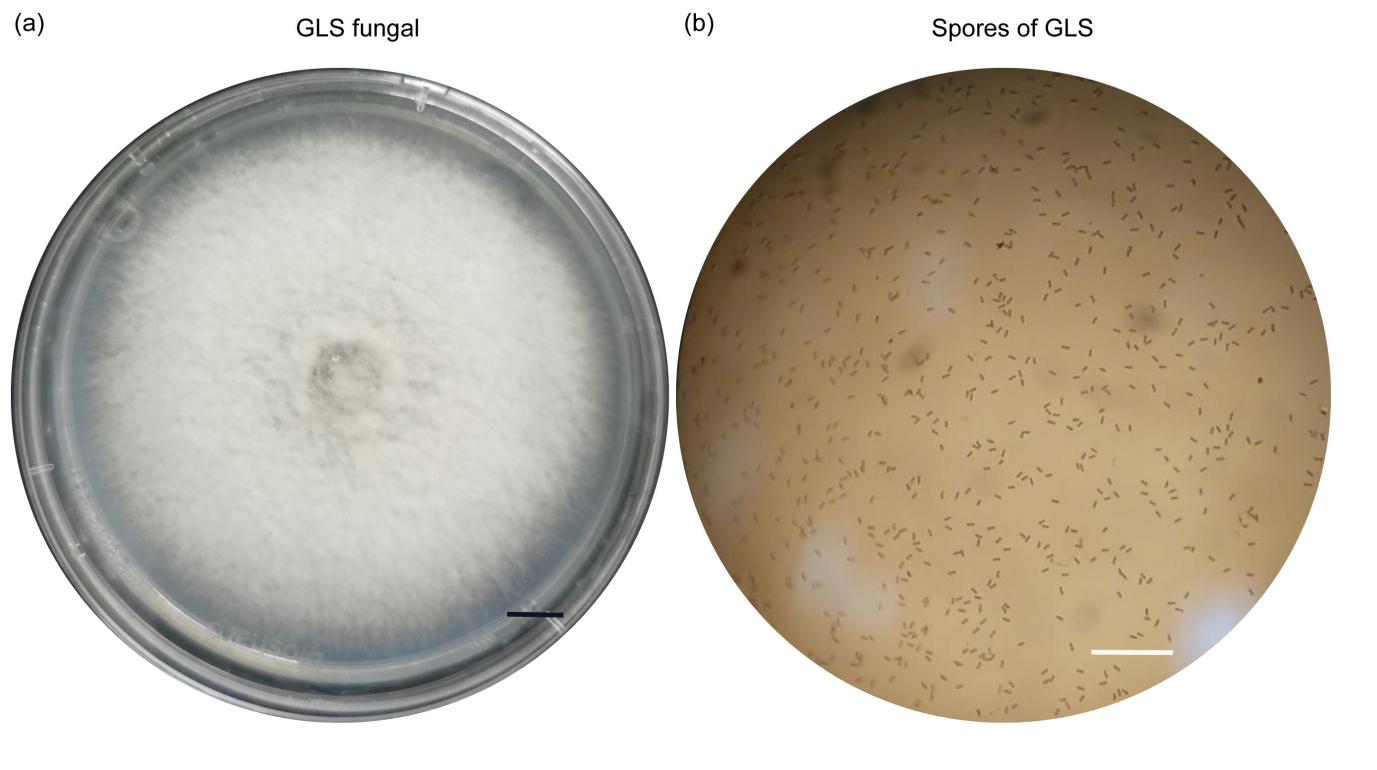


**FIGURE S5** The photos of GLS fungal and its spores cultured for five days. (a) GLS fungal. Bar, 10 cm. (b) Spores of GLS. Bar, 100 μm. GLS, Glomerella leaf spot.
