# Supplementary material for: The m6A reader MhYTP2 negatively modulates apple Glomerella leaf spot resistance by binding to and degrading MdRGA2L mRNA
Source: Mol Plant Pathol. 2023 Jun 27;24(10):1287–99. doi: 10.1111/mpp.13370 (PMC10502827; doi:10.1111/mpp.13370)
Supplement: Supplementary file 8 — TEXT S1. RNA probe and primers used [file MPP-24-1287-s002.docx]

**Text S1**

**Digoxin labeled RNA oligo-nucleotides of *MdRGA2L* for EMSA.**

agaucucagucagugucuuagucuuguggaauugccuagagacauuaaa

**Sequences of primers for quantitative real-time PCR.**

| **Gene** | **Primer** | **Sequence (5**′**–3**′**)** |
| --- | --- | --- |
| *MdRGA2L* | RT-*MdRGA2L*-S | GTTTGGAGGATGTTGCGTTT |
|  | RT-*MdRGA2L*-A | CAACTACGACGCTCCTGACA |
| *MdICS1* | RT-*MdICS1*-S | CAGCAAATTGAAGCAATCGACTGG |
|  | RT-*MdICS1*-A | CTGGAGACGTATAATTCCGGAGGA |
| *MdPR1* | RT-*MdPR1*-S | GGCTCAGTCCTTATCCAATCCTC |
|  | RT-*MdPR1*-A | GCCTGCTACTTTGTCATCCCACG |
| *MdMDH* | RT-*MdMDH*-S | CGTGATTGGGTACTTGGAAC |
|  | RT-*MdMDH*-A | TGGCAAGTGACTGGGAATGA |
| *MdActin* | RT-*MdActin*-S | TGACCGAATGAGCAAGGAAATTACT |
|  | RT-*MdActin*-A | TACTCAGCTTTGGCAATCCACATC |
| *MD07G1016200* | RT-*MD07G1016200*-S | GTTTGGAGGATGTTGCGTTT |
|  | RT-*MD07G1016200*-A | CAACTACGACGCTCCTGACA |
| *MD00G1028200* | RT-*MD00G1028200*-S | TGGAGTGCAGATCCAAACTG |
|  | RT-*MD00G1028200*-A | CCCCGTCCTCAGTGTTTTTA |
| *MD00G1027800* | RT-*MD00G1027800*-S | AGCGTCTCCTGACCTCTCAA |
|  | RT-*MD00G1027800*-A | ACCGAGTTGGATGTGTGTCA |
| *MD00G1020300* | RT-*MD00G1020300*-S | CAACCCTCTCCTGAAATGGA |
|  | RT-*MD00G1020300*-A | CTGCATATGTACGGCACCAC |
| *MD07G1019800* | RT-*MD07G1019800*-S | AAGTTGGTTGGATGCGTTTC |
|  | RT-*MD07G1019800*-A | CCAATCGAATCTCGTCCCTA |
| *MD08G1230200* | RT-*MD08G1230200*-S | TAGCCCCAAGGTTTTCAATG |
|  | RT-*MD08G1230200*-A | CACATCTTTCCACTCGCGTA |
| *MD00G1027700* | RT-*MD00G1027700*-S | TAGGGATCAGCGTCCTCTGT |
|  | RT-*MD00G1027700*-A | TTCTCGAGGATTCCACAACC |
| *MD07G1246200* | RT-*MD07G1246200*-S | TCAAGGAGGGTTTCATGAGG |
|  | RT-*MD07G1246200*-A | AGCATTGTCGCTCTCCAAGT |
| *MD07G1246300* | RT-*MD07G1246300*-S | CTTCGGCGAATTTTCTCTTG |
|  | RT-*MD07G1246300*-A | CCAGCTCCTAACAGCGATTC |
| *MD02G1054500* | RT-*MD02G1054500*-S | TGGCCAACATCTTCAAATCA |
|  | RT-*MD02G1054500*-A | CCAACCATGCAAACAACATC |
| *MD15G1180000* | RT-*MD15G1180000*-S | CTGAAAAATCCCCCACTCAA |
|  | RT-*MD15G1180000*-A | GCAAATGCTTTTGTGCTGAA |
| *MD01G1189200* | RT-*MD01G1189200*-S | TCAGATGTCAATGGCTGCTC |
|  | RT-*MD01G1189200*-A | AAGATCGTCGCCTTTTCTGA |
| *MD09G1186700* | RT-*MD09G1186700*-S | CCCTTCCATTTCTGCTCTTG |
|  | RT-*MD09G1186700*-A | ACAAGCCCCGTAAGATTGTG |
| *MD00G1028000* | RT-*MD00G1028000*-S | CCTTGTGGTGAGCGTTCATA |
|  | RT-*MD00G1028000*-A | CATTCCCTCCAAGATCCAAA |
| *MD07G1244600* | RT-*MD07G1244600*-S | GGAAACCCCTGACTTCACAA |
|  | RT-*MD07G1244600*-A | ATCTCAAGGCTCACGCAGTT |
| *MD17G1030800* | RT-*MD17G1030800*-S | AGTGAAATCGTTGGGACGAC |
|  | RT-*MD17G1030800*-A | TTTTCTTGCAACCTCCATCC |
| *MD07G1260000* | RT-*MD07G1260000*-S | GTGCTCCCCATTTTCTACGA |
|  | RT-*MD07G1260000*-A | AAACTGGTGGCCGTTGTAAG |
| *MD02G1039200* | RT-*MD02G1039200*-S | ATTTTGCCGAAGCATTTCAG |
|  | RT-*MD02G1039200*-A | TGCTTCGTGCCTATTGTCAG |
| *MD16G1068200* | RT-*MD16G1068200*-S | CTTCGTCAACATCAGGCTCA |
|  | RT-*MD16G1068200*-A | GAACGGCAGGGATGAATAGA |
| *MD07G1024400* | RT-*MD07G1024400*-S | AGGCTCCCTAGCTTTCCAAG |
|  | RT-*MD07G1024400*-A | CGGCTTCATAAATTGCGTCT |
| *MD07G1245600* | RT-*MD07G1245600*-S | CTCACGGGATTGAGGTGTTT |
|  | RT-*MD07G1245600*-A | TCCACCCAAGAAAAGCATTC |
| *MD10G1297400* | RT-*MD10G1297400*-S | AGTTTTGCGCAATCGTTTCT |
|  | RT-*MD10G1297400*-A | GTTTTGTTCAGCACCCGAAT |
| *MD05G1317300* | RT-*MD05G1317300*-S | ACAAAGGCTGGAATCAATGC |
|  | RT-*MD05G1317300*-A | AATGCCGGCAAAACTAATTG |
| *MD15G1179600* | RT-*MD15G1179600*-S | TACAACATGCCAACCCTCAA |
|  | RT-*MD15G1179600*-A | TCTTCATTCATTCCCCAAGC |
| *MD12G1248800* | RT-*MD12G1248800*-S | AGAGGCGGATATTCCTCGAT |
|  | RT-*MD12G1248800*-A | CTCTGCCCATATCTCGAAGC |
| *MD07G1261100* | RT-*MD07G1261100*-S | CCGCTGGAGAAATCCAATTA |
|  | RT-*MD07G1261100*-A | TCACCATCAGCAGCTCATTC |
| *MD02G1026100* | RT-*MD02G1026100*-S | ATTACATGTGGCCAAGCACA |
|  | RT-*MD02G1026100*-A | TACTTGTAGCATCGCGAACG |
| *MD07G1014900* | RT-*MD07G1014900*-S | GGAGGATGTTGCGTTTGAAT |
|  | RT-*MD07G1014900*-A | CAAAGTTGGCACTTCCCATT |
| *MD07G1023800* | RT-*MD07G1023800*-S | TTCTACGCTTTGGAGCCACT |
|  | RT-*MD07G1023800*-A | AAGAAATGGCGGAAGGAGTT |
| *MD02G1040900* | RT-*MD02G1040900*-S | AAAGAACCATGGCAGAGTGG |
|  | RT-*MD02G1040900*-A | ACCGTTACAAGGCATCGTTC |
| *MD07G1015300* | RT-*MD07G1015300*-S | TACTTGGAGGGCAACATTCC |
|  | RT-*MD07G1015300*-A | CTGGTGGGTTGGTTTGTTCT |
| *MD03G1280000* | RT-*MD03G1280000*-S | TGCGACATGTTAAGCGAAAG |
|  | RT-*MD03G1280000*-A | TCTGGCCGGTTACTTGGTAG |
| *MD02G1260000* | RT-*MD02G1260000*-S | GGCCACACTCAAACAATGTG |
|  | RT-*MD02G1260000*-A | TCTCGCGGTTAAAATTGGAC |
| *MD04G1011700* | RT-*MD04G1011700*-S | GCGGAATCAAGATGGTGTCT |
|  | RT-*MD04G1011700*-A | TGAGTAAGAGCCTCCCTCCA |
| *MD02G1038400* | RT-*MD02G1038400*-S | AAGCAGCTCCAACTTTTCCA |
|  | RT-*MD02G1038400*-A | GATGAAGAGGAGGCTTCGTG |
| *MD07G1017400* | RT-*MD07G1017400*-S | TTGTGATGGGTTGACTGGAA |
|  | RT-*MD07G1017400*-A | TGACACCACATGCCTCAAAT |
| *MD07G1019600* | RT-*MD07G1019600*-S | TGGGTGATGCATGGTTCTTA |
|  | RT-*MD07G1019600*-A | GCAGCGCATCATTTCTGATA |
| *MD07G1019700* | RT-*MD07G1019700*-S | TTTTCTTGCAACCTCCATCC |
|  | RT-*MD07G1019700*-A | GGGTGGGGGAAAAGGTAGTA |
| *MD02G1054400* | RT-*MD02G1054400*-S | AGTTTGGGAGGGTTCCAAGT |
|  | RT-*MD02G1054400*-A | ATTCTTGCAGCCTGTGAGGT |
| *MD07G1024100* | RT-*MD07G1024100*-S | CGCACACTCAAAACAGTGCT |
|  | RT-*MD07G1024100*-A | TGTGGGATGTGTGCAATCTT |
| *MD03G1280200* | RT-*MD03G1280200*-S | CAGCAGCGGCAATAGTATGA |
|  | RT-*MD03G1280200*-A | GAAAGACCGGGAAAAGAAGG |
| *MD00G1021100* | RT-*MD00G1021100*-S | GGCACTTTGGATCGAGGTTA |
|  | RT-*MD00G1021100*-A | AGATCGCATCAGCTCCACTT |
| *MD15G1182500* | RT-*MD15G1182500*-S | GCCAGGTTTGGACAAGTCAT |
|  | RT-*MD15G1182500*-A | ACTCAACTTCCTCGCCTTCA |
| *MD02G1053600* | RT-*MD02G1053600*-S | ACAGGTCCAAATCCGTTCAG |
|  | RT-*MD02G1053600*-A | ATTTGGCTCTTTGTGGAACG |
| *MD07G1260600* | RT-*MD07G1260600*-S | TTCGCTGAAGCTTTTGATCG |
|  | RT-*MD07G1260600*-A | AATTTCCTCAATAAGCTTTGCAT |
| *MD07G1243000* | RT-*MD07G1243000*-S | GCCTCAGAAATCACCCAAAA |
|  | RT-*MD07G1243000*-A | CCCACCAAAGCAATCTCATT |
| *MD07G1244000* | RT-*MD07G1244000*-S | TGTCGTTCACCGCTGTAGTC |
|  | RT-*MD07G1244000*-A | TCAGTTACGGGGAACACTCC |
| *MD07G1023700* | RT-*MD07G1023700*-S | ATTTGAGGCATGTGGTGTCA |
|  | RT-*MD07G1023700*-A | AACCAACTCGCAAACCTCAC |
| *MD07G1242100* | RT-*MD07G1242100*-S | CTATACGCGTGTGGGAAGGT |
|  | RT-*MD07G1242100*-A | TTCAGTTGCCTGGTCTTCCT |
| *MD00G1026000* | RT-*MD00G1026000*-S | CACGAAGCCTCCTCTTCATC |
|  | RT-*MD00G1026000*-A | AGATCCTCGACCCTTCGATT |
| *MD17G1056800* | RT-*MD17G1056800*-S | GCCTTCAGCAATCCAGAGTC |
|  | RT-*MD17G1056800*-A | GCCGGAAGAATGTTACGTGT |
| *MD07G1243500* | RT-*MD07G1243500*-S | CTCCGAGCACACAATTCAGA |
|  | RT-*MD07G1243500*-A | AATCAGGCGTCCACAGAATC |
| *MD00G1011700* | RT-*MD00G1011700*-S | TGCCACTAGCCCTTGAAGTT |
|  | RT-*MD00G1011700*-A | TTGCAGAAAATCCACATCCA |
| *MD17G1217000* | RT-*MD17G1217000*-S | TGGACACTCGCCAAATTACA |
|  | RT-*MD17G1217000*-A | AACATCCGTCAGTCCTCCAC |
| *MD02G1031800* | RT-*MD02G1031800*-S | TAGTTGGCATGCATTTGGAA |
|  | RT-*MD02G1031800*-A | ATCTAGCCCGTCAAAGCTCA |
| *MD09G1039600* | RT-*MD09G1039600*-S | ATCTCCCCCACCCTTGATAC |
|  | RT-*MD09G1039600*-A | TGGTAGTGCTCCTGCTCCTT |
| *MD05G1316800* | RT-*MD05G1316800*-S | CGGGGACTGAGACATTCATT |
|  | RT-*MD05G1316800*-A | AGGAAAAAGGGAAAGGCTGA |
| *MD02G1052800* | RT-*MD02G1052800*-S | CACGAAGCCTCCTCTTCATC |
|  | RT-*MD02G1052800*-A | TTGCCTGGAGCAGTTCTTTT |
